# Acanthus Condom Catheter: A Reusable and Adjustable Silicone Male External Catheter With Pressure-Sensitive Silicone Adhesive for Urinary Drainage

**DOI:** 10.7759/cureus.69482

**Published:** 2024-09-15

**Authors:** Venkata Buddharaju, David Shelton

**Affiliations:** 1 Pulmonary and Critical Care Medicine, Thorek Memorial Hospital, Chicago, USA; 2 Engineering, Skinister Medical, Woodland, USA

**Keywords:** adjustable condom catheter, catheter slippage, cauti, cauti prevention, condom catheter, male external catheter, reusable, silicone adhesive, urine incontinence, urine leak

## Abstract

Condom catheters are also called external urinary collection devices to collect urine and monitor urine output in hospitalized and other patients with urinary incontinence. They play an important role in reducing catheter-associated urinary tract infections by using invasive indwelling catheters that are placed inside the bladder. Currently, male external catheters come with or without adhesives. Major problems with current condom catheters are finding the right size to fit, excessive slippage needing to replace too many, urine leak, skin irritation, and damage. In some cases, it's a common practice to wrap adhesive tape around the catheter and penis to keep the catheter in place which can cause skin injury. The Acanthus condom catheter is an expandable silicone male external catheter designed and developed to reduce excessive catheter slippage and keep the catheter longer. It works by applying silicone adhesive to the inner catheter wall just below the catheter rim and applying gentle pressure after the placement. It can be adjusted after the placement and can be kept in place by applying gentle pressure around the catheter to prevent slippage. After laboratory testing of the catheter showed it was able to withstand 250% more pressure and 187% higher tensile forces, we have retrospectively reviewed a nursing survey of 14 hospitalized patients using the Acanthus condom catheter for urinary drainage. The mean age was 89 years. A total of 55 evaluations were collected, of which 46 were day shift and nine were night shift responses for a total of 44 catheter days. Results showed that the catheter was easy to use (100%). There was no skin irritation or damage during the application or removal (100%). The catheter was able to drain urine and stayed in place most of the time (89%) except in a few instances it came out, especially during the night shift, but the nurse was able to place it back. There was one instance of urine leak (1.8%). Nurses felt that the catheter was better than existing products in 95% of responses. In conclusion, the Acanthus condom catheter was easy to use, can be reused for up to three days, and was safe on the skin; most importantly, nurses were able to adjust the catheter when they noticed it to be slipping and kept it in place using gentle finger pressure or adding few additional drops of silicone adhesive on the inner catheter wall below the rim and applying brief pressure around the catheter. One size (medium-large) fits most of the patients in the study due to elasticity and stretch dimensions, which eliminated the problem of finding the right size to use.

## Introduction

Male external catheters also called condom catheters (often referred to as Texas-style catheters) have been widely used in male patients with no urinary obstruction to drain urine in hospitalized and other patients with urine incontinence. They are also used to measure urine output as part of intake and output measurements. Infection control teams monitor and recommend replacing indwelling urinary catheters with male external catheters or urinals to reduce the risk of catheter-associated urinary tract infections (CAUTI), a reportable hospital-acquired condition or infection. Evidence-based strategies are implemented to prevent CAUTI in the hospital setting [1,2].

Condom catheters come as self-adhering adhesives in multiple size options that are placed over the penile shaft and connected to the urinary drainage bag to the gravity drain. Frequent slippage (especially in agitated and moving patients) requiring to replace with a new one is a common problem, and sometimes, an additional adhesive tape is wrapped around the penis and the catheter risking skin injury. Frequent placement of adhesive catheters can result in skin irritation and damage and can be cost-prohibitive. Selecting the correct size can be challenging, especially since placing a smaller than usual size may result in skin constriction and damage; on the other hand, placing a larger size can result in slippage and urine leaks. Recently, pouch-type devices came, but they require constant suction to work with the potential for urine leak due to ineffective seal. 

The Acanthus condom catheter is a specially designed silicone male external catheter with differential wall thickness that is stretchable to accommodate the size and shape after placement. After the application of a pressure-sensitive silicone adhesive (Skinister Medical Products) to the inner catheter wall under the catheter rim, it forms a strong bond with the skin and keeps the catheter in place during use. As the name implies, pressure-sensitive adhesive continues to form an adhesive bond to the skin, and the adhesion slowly wears off over time due to skin perspiration. If the catheter is noticed to be slipping, applying gentle pressure or a few adhesive drops on the inner catheter wall below the catheter rim can restore adhesion and keep the catheter secure. Reducing frequent slippage decreases the need to replace with a new one and can potentially reduce cost and prioritize nursing time more effectively.

We retrospectively reviewed 14 hospitalized patients who used the Acanthus condom catheter. Nursing staff were asked to complete the survey questions. The responses were presented as a percentage of yes or no answers to the questions. The study was done as part of a quality improvement project and was approved by the Department of Nursing, Thorek Memorial Hospital, Chicago, Illinois, United States.

## Materials and methods

Acanthus condom catheter

The Acanthus condom catheter was tested for pressure and tensile strength with and without the application of adhesive on the inner wall below the catheter rim.

Pressure Test 

Test setup: The Acanthus condom catheter was applied to an aluminum mandrel. A size medium-large catheter was used, which is intended for a penis diameter of 26-36 mm, and a mandrel diameter of 31.75 mm was selected, which is the middle of the intended size range. The condom was pressurized with air until failure occurred. The test was performed with and without the Skinister Medical silicone adhesive applied to the inner catheter wall below the catheter rim.

**Figure 1:**
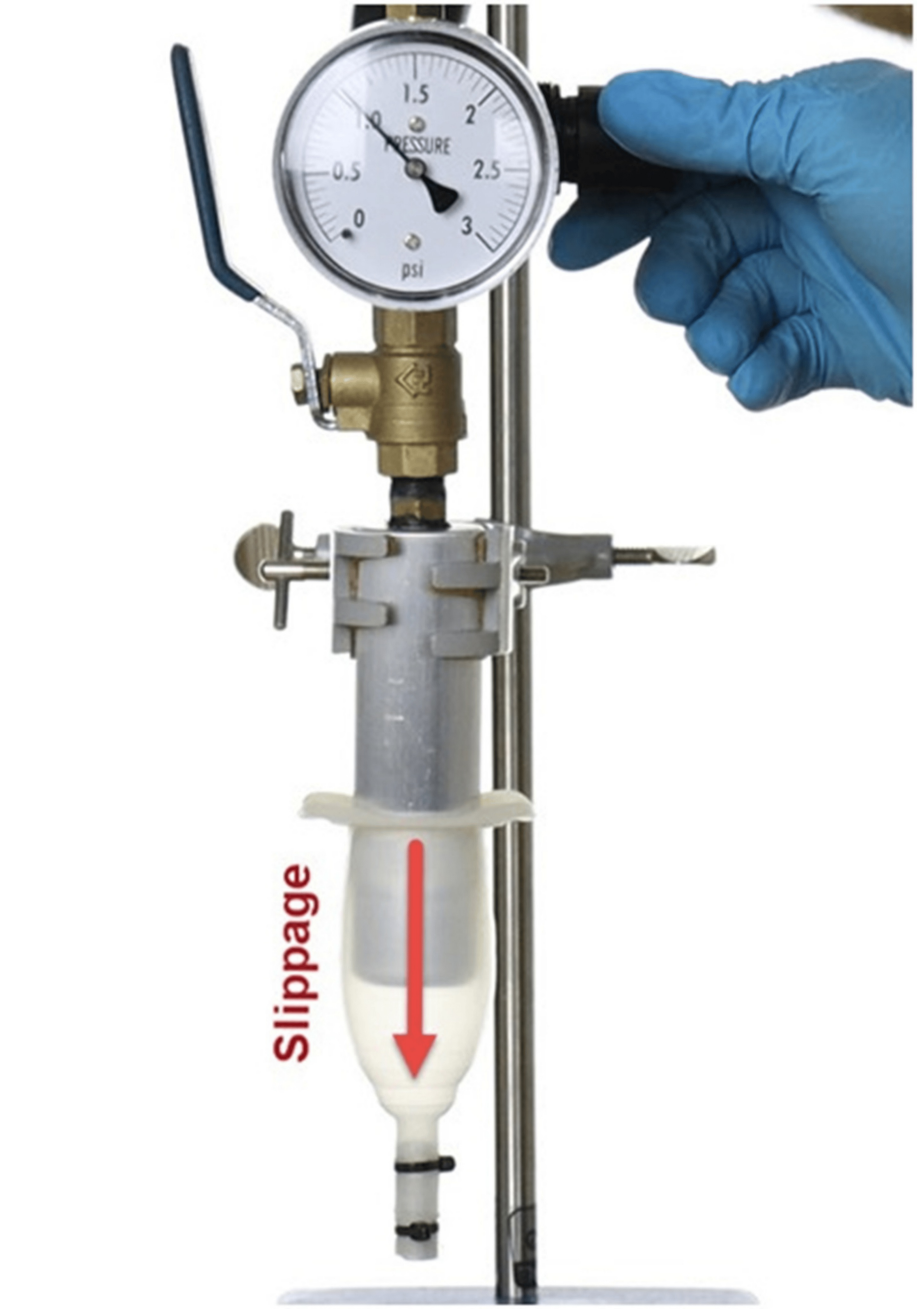
Acanthus condom catheter pressure test without adhesive

Figure 1 shows the Acanthus condom catheter pressure test without adhesive. At a pressure of 1 psi, the catheter began to slowly slide off the mandrel. Failure was more pronounced at higher pressures, with the condom catheter ejecting off the mandrel with high velocity.

**Figure 2:**
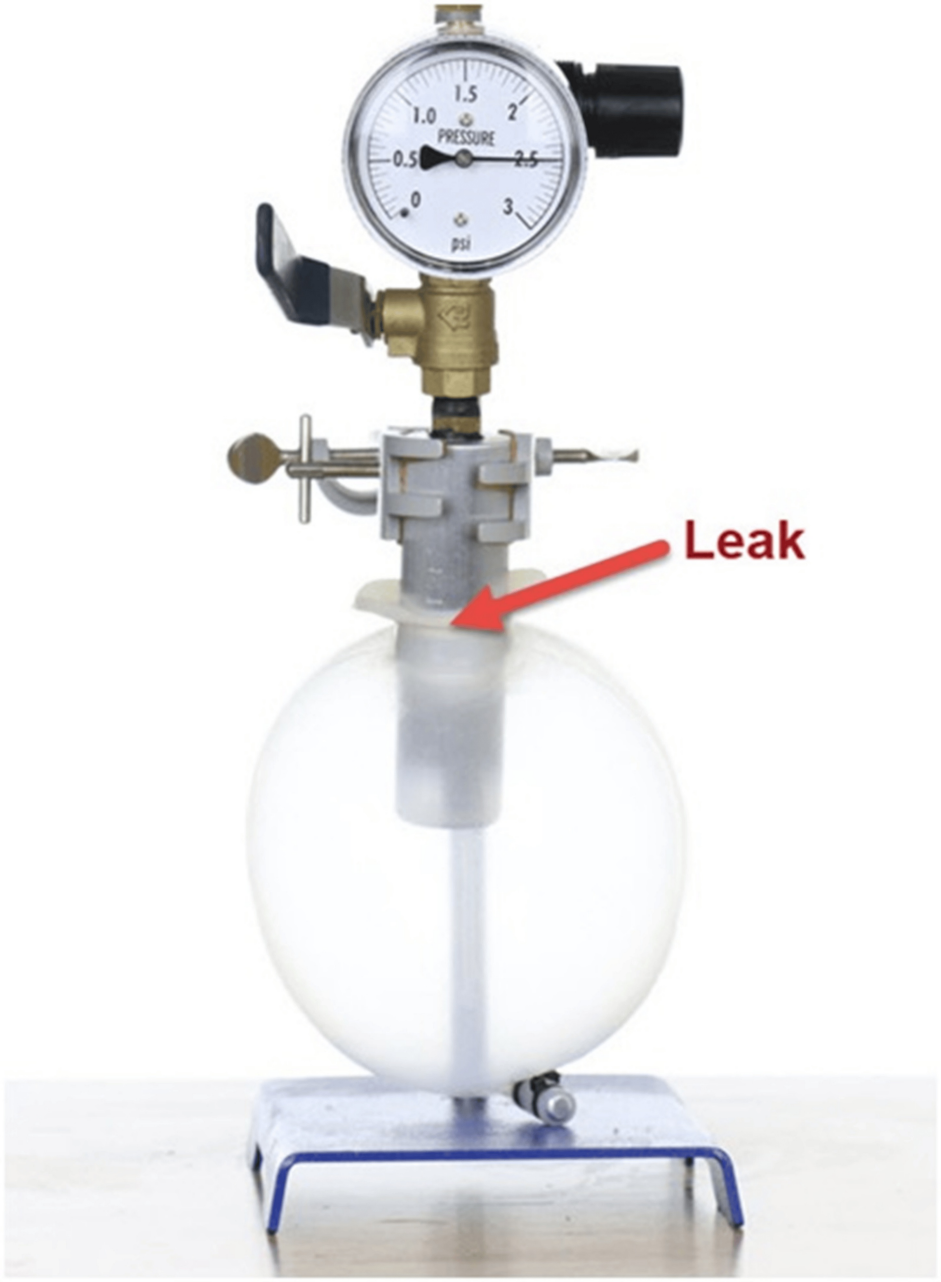
Acanthus condom catheter pressure test with adhesive

Figure 2 shows the Acanthus condom catheter pressure test with adhesive. As the pressure increased, the tip of the condom catheter ballooned and took on a spherical shape. This caused the adhesive forces to transition from in-line shear to high-angle peel. At a pressure of 2.5 psi, the adhesive gradually began to fail in the peel. The peel progressed slowly until a leak developed at the base of the catheter.

The adhesive was observed to have excellent resealing capabilities; pressing on the adhesive area successfully stopped the leak, and the experiment was repeated with similar results.

Tensile Test 

Test setup: The Acanthus condom catheter was applied over a water snake toy, which is representative of a flaccid penis. Tensile forces were applied by a Mark-10 motorized test stand at a constant speed of 10 inches per minute until slippage occurred. The test was performed with and without the Skinister Medical silicone adhesive.

**Figure 3:**
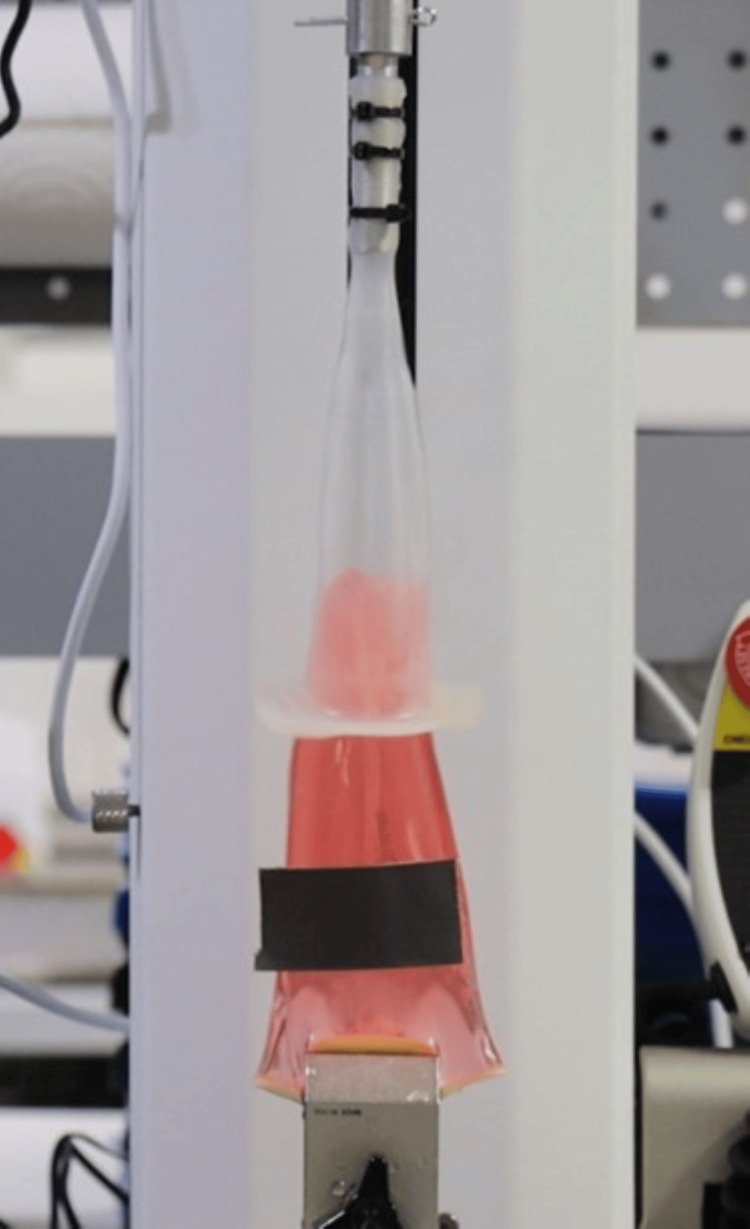
Acanthus condom catheter tensile test without adhesive

Figure 3 shows the Acanthus condom catheter tensile test without adhesive.

**Figure 4:**
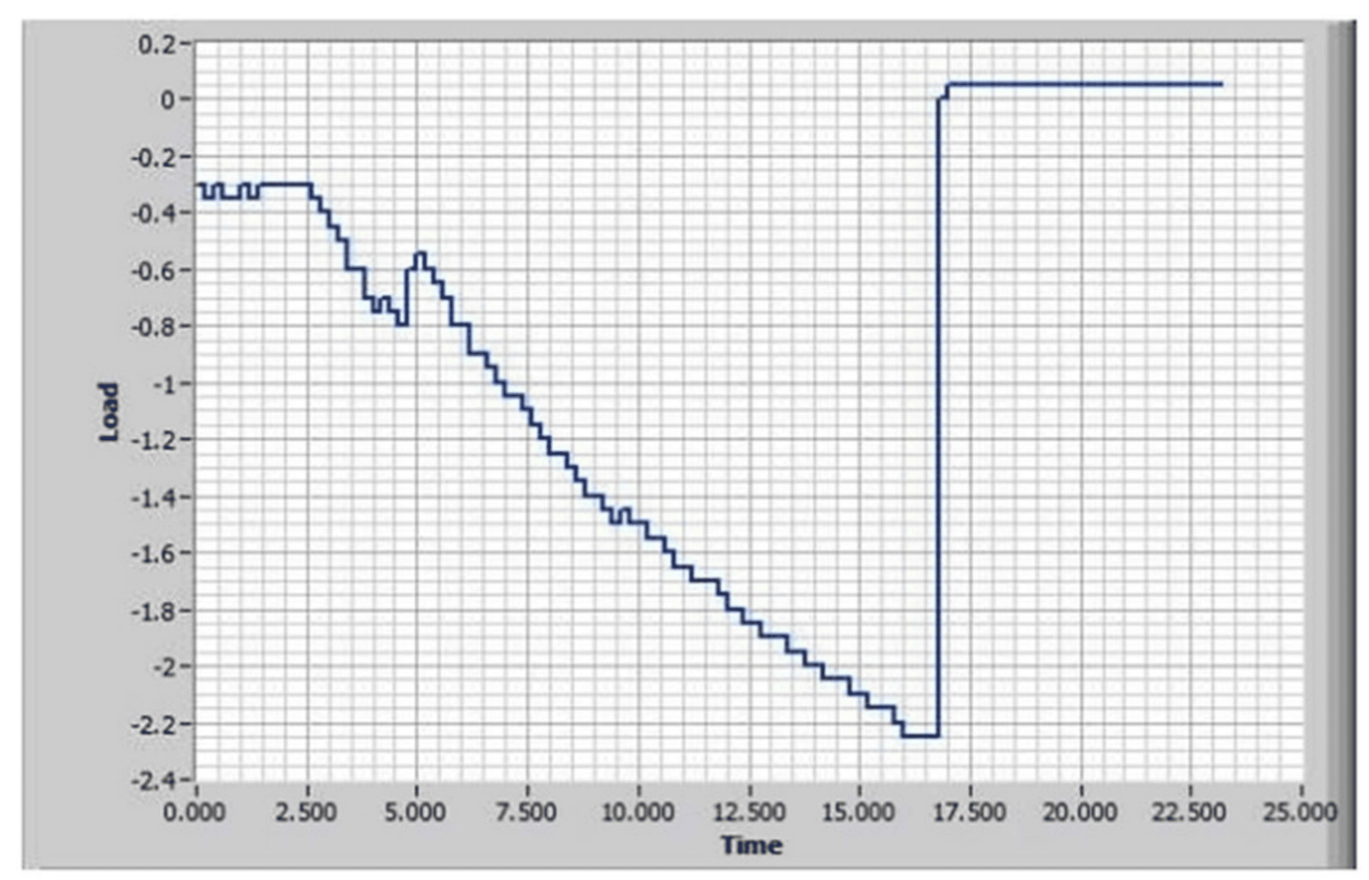
Acanthus condom catheter tensile test graph without adhesive

Figure 4 shows the Acanthus condom catheter tensile test graph without adhesive where the condom catheter slipped off at a force of 2.25 pounds. 

**Figure 5:**
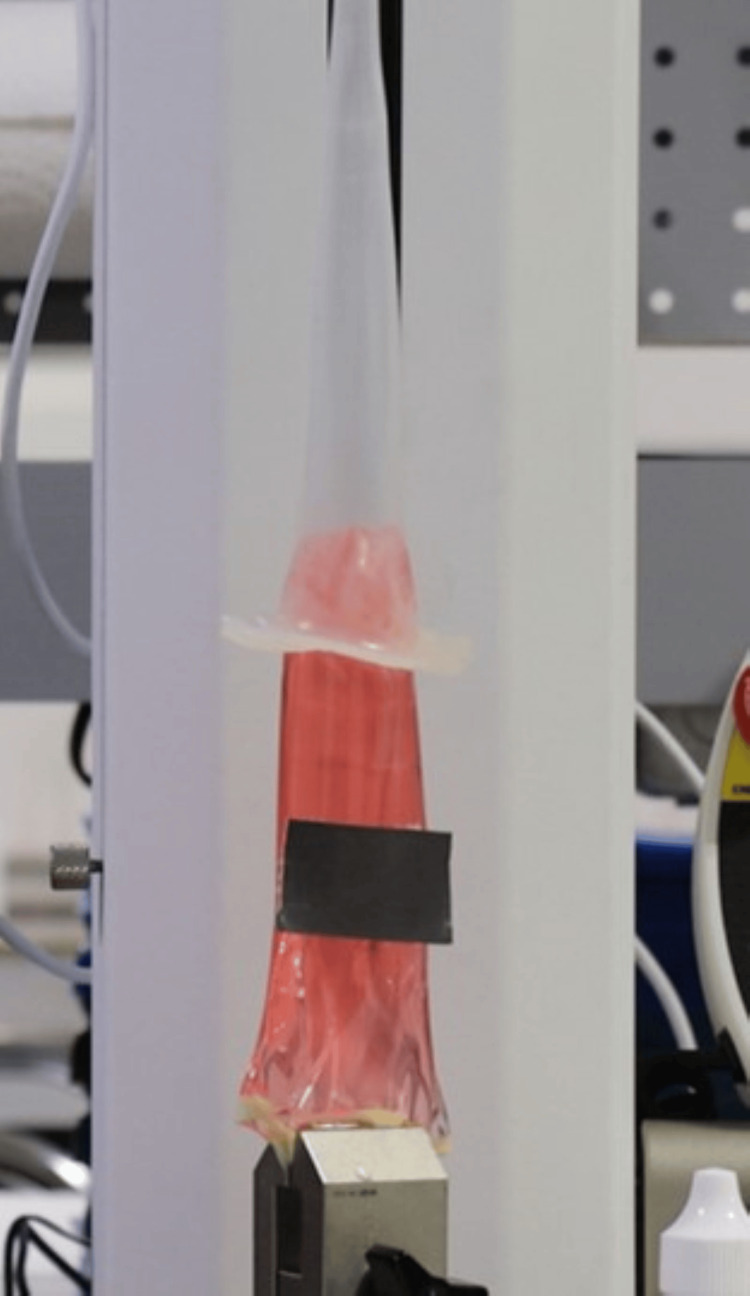
Acanthus condom catheter tensile test with adhesive

Figure 5 shows the Acanthus condom catheter tensile test with adhesive.

**Figure 6:**
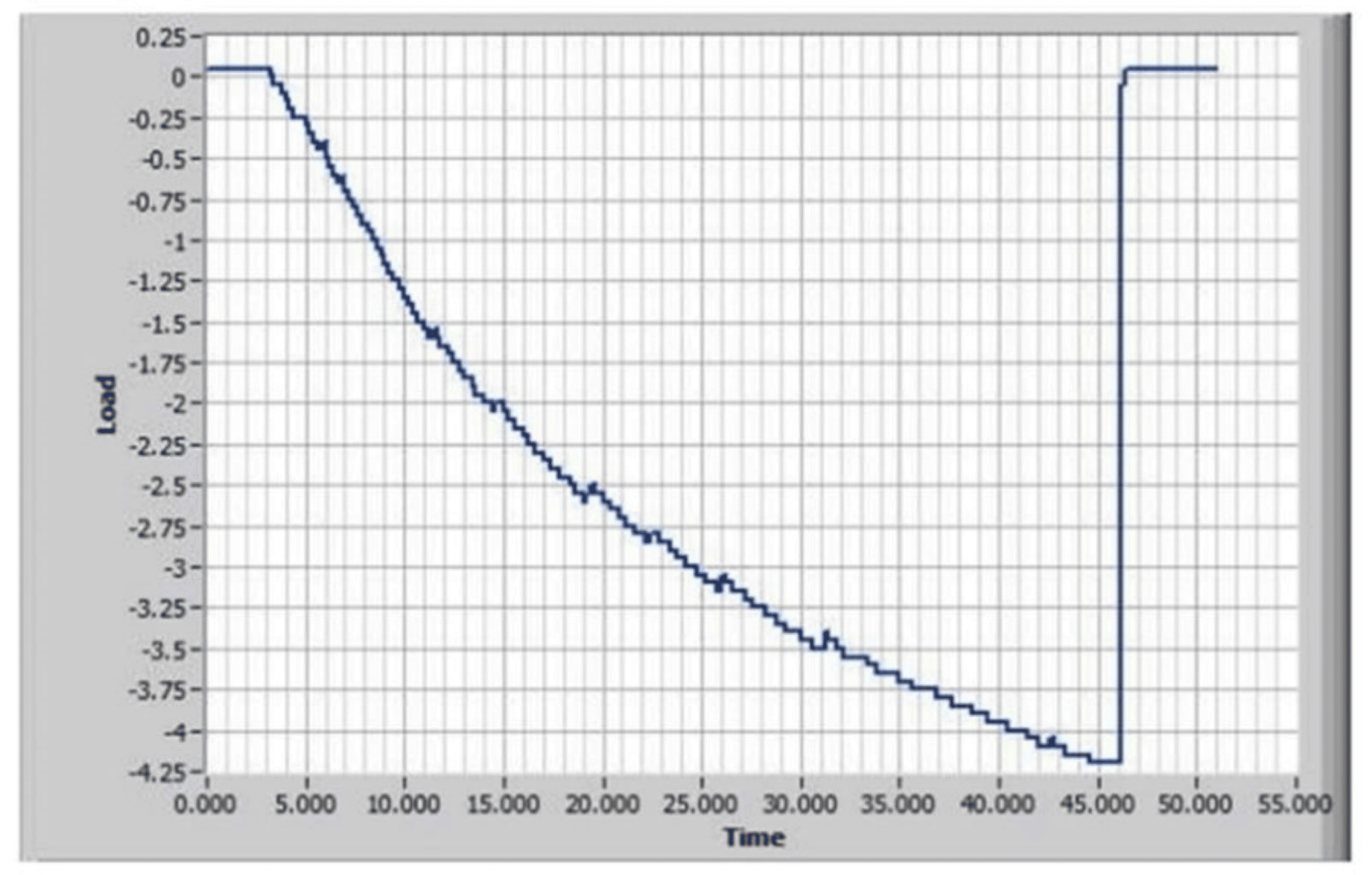
Acanthus condom catheter tensile test graph with adhesive

Figure 6 shows the Acanthus condom catheter tensile test graph with adhesive. The Skinister Medical silicone adhesive was applied to the inner catheter wall below the rim in accordance with Acanthus Instructions for Use. The condom catheter slipped off at a force of 4.2 pounds.

Conclusion of the Laboratory Testing of the Acanthus Condom Catheter With and Without Pressure-Sensitive Silicone Adhesive (Skinister Medical Products)

Without adhesive, the condom catheter was consistently ejected from the mandrel at very low pressures and tensile forces. After the Skinister Medical silicone adhesive was applied to the inner wall of the catheter below the catheter rim, it was able to withstand 250% more pressure and 187% higher tensile force.

Additional performance gains are anticipated if the adhesive is applied to a greater surface area, but this has not yet been tested. Furthermore, the adhesive was found to have excellent re-sealing capabilities. After a leak develops, significant adhesion may be restored by applying moderate pressure to the area with a fingertip. These laboratory results are consistent with clinical evaluations in which the Skinister Medical silicone adhesive was found to decrease instances of leaks and slippage.

**Figure 7:**
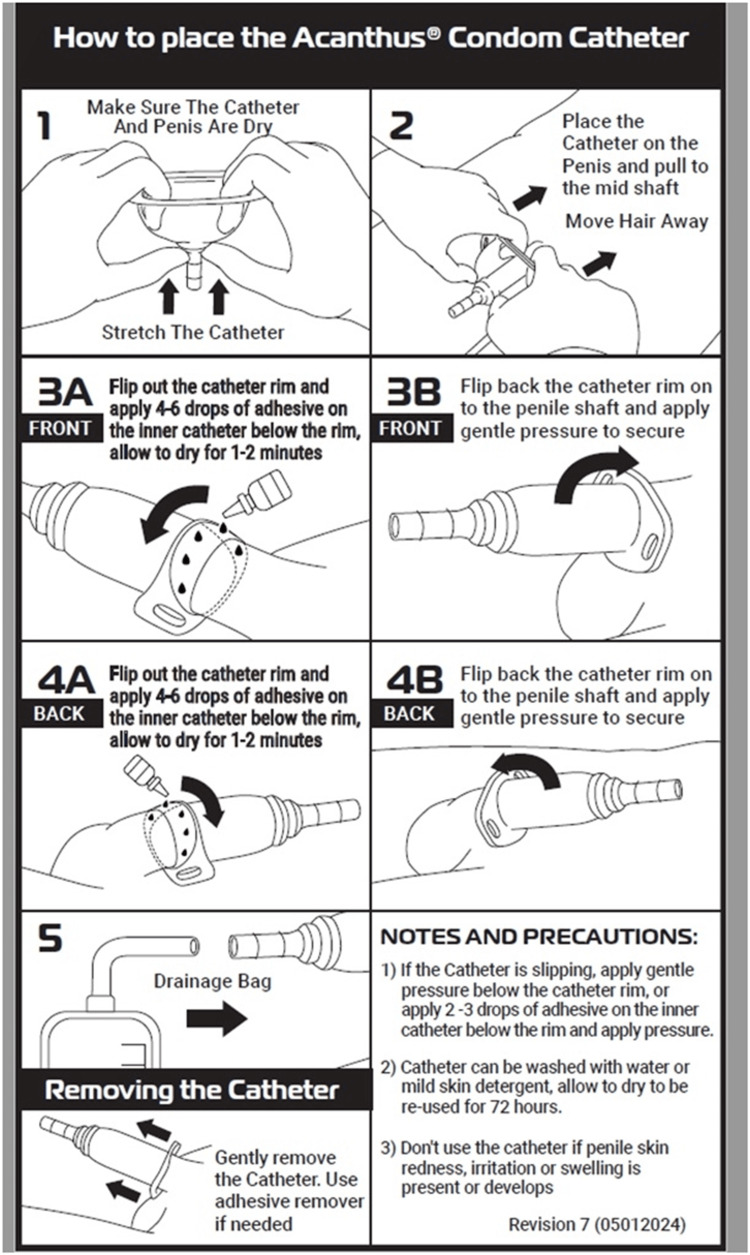
Instructions on how to use the Acanthus condom catheter

Instructions on how to use the Acanthus condom catheter were given to the nursing staff and certified nursing assistants (Figure 7) along with an instructional video (Video 1).

**Video 1:** Acanthus condom catheter video instructions

Nurses and certified nurse assistants were asked to complete the survey questionnaire (Appendices) to evaluate the Acanthus condom catheter use on patients during their respective shifts. All responses were reviewed along with the patient's brief history and diagnosis. The data was compiled in a spreadsheet and tabulated as a percentage of yes or no answers along with their comments. The percentage of all responses was summarized as well. 

The Acanthus condom catheter was supplied in a bag with a 3-ml pressure-sensitive silicone adhesive bottle that lasts for 10 applications and/or three days of use. A total of 14 hospitalized patients who used the Acanthus condom catheter for urinary drainage from January 1, 2024, to July 31, 2024, were reviewed. Cases were identified as numbers from 1 to 14 with a brief description of the patient's diagnosis. The mean age and percentage of responses to the questions in the survey were calculated. Nursing comments along with the results of all patient evaluations were tabulated and reported.

## Results

Clinical case series 

Patient 1

Patient 1 is a 57-year-old male with a history of hypertension and polysubstance abuse who presented due to mental status changes, confusion, agitation, and restlessness diagnosed as opiate withdrawal. He was managed with noninvasive ventilation during the hospital course for acute hypoxic respiratory failure. The Acanthus condom catheter was used during the hospital course for urine incontinence and to monitor intake and output.

Patient 2

Patient 2 is an 88-year-old male with a history of hypertension, chronic kidney disease, and dementia admitted due to an unresponsive state and mental status changes. Fluids and electrolytes were managed, and urine output was measured with the Acanthus condom catheter.

Patient 3

Patient 3 is a 67-year-old male with a history of congestive heart failure and atrial fibrillation admitted due to gastrointestinal bleeding with a low hemoglobin of 4.7 gm. Strict intake and output were monitored along with urine output using the Acanthus condom catheter.

Patient 4

Patient 4 is a 65-year-old male with a history of chronic obstructive pulmonary disease (COPD) and chronic alcohol use admitted due to alcohol withdrawal. He was confused, agitated, and restless with tachycardia and had delirium. Fluids and electrolytes were closely monitored. Urine output was measured and monitored using the Acanthus condom catheter.

Patient 5

Patient 5 is a 78-year-old male with multiple medical problems of diabetes, chronic kidney disease, stroke, and heart failure admitted due to severe hypernatremia. An indwelling urinary catheter was removed on day 1, and the Acanthus condom catheter was placed for urine output monitoring.

Patient 6

Patient 6 is a 59-year-old male admitted due to abdominal pain. He underwent exploratory laparotomy and sigmoid colon resection for cancer. Post-op, urine output was monitored with the Acanthus condom catheter.

Patient 7

Patient 7 is an 87-year-old male with a history of congestive heart failure admitted due to acute hypoxic respiratory failure managed with noninvasive ventilation, oxygen, and Lasix. Urine output was monitored with the Acanthus condom catheter.

Patient 8

Patient 8 is a 76-year-old male with a history of hypertension, congestive heart failure, renal failure, and diabetes admitted due to gastrointestinal bleeding. He received a blood transfusion and fluids. Urine output was measured and monitored with the Acanthus condom catheter.

Patient 9

Patient 9 is an 89-year-old male with a history of colon cancer with colostomy who developed left pneumothorax post-Portacath insertion of the left subclavian vein. He had pneumonia and septic shock and was given IV fluids with saline bolus. Antibiotics were administered, and urine output was monitored with the Acanthus condom catheter.

Patient 10

Patient 10 is a 65-year-old male with a history of alcohol abuse, admitted due to alcohol withdrawal syndrome and aspiration pneumonia, severe hypoxemia requiring noninvasive ventilation, agitation, restlessness, and confusion with moderate encephalopathy. Urine output was monitored during the hospital course using the Acanthus condom catheter.

Patient 11

Patient 11 is a 61-year-old male with a history of chronic hypoxic respiratory failure, hypertension, and encephalopathy admitted due to pneumonia. The Acanthus condom catheter was placed for urine output and continence. 

Patient 12

Patient 12 is an 81-year-old male with a history of dementia, diabetes, and pancreatic cancer s/p resection, admitted due to hyponatremia and hypoxic respiratory failure requiring oxygen and normal saline. The Acanthus condom catheter was placed for urine monitoring.

Patient 13

Patient 13 is an 81-year-old male with a history of dementia and diabetes readmitted after two weeks due to severe hypercapnic respiratory failure and unresponsive state, atrial fibrillation, and rapid ventricular response managed with noninvasive ventilation and intravenous diltiazem drip for heart rate control. The Acanthus condom catheter was applied for urine output monitoring. 

Patient 14

Patient 14 is an 89-year-old male with a history of colon cancer readmitted after two months due to weakness, vomiting, and atrial fibrillation with rapid ventricular response managed with intravenous diltiazem drip and antibiotics for sepsis. The Acanthus condom catheter was applied for urine output measurement. 

A total of 14 patients with two readmissions used the Acanthus condom catheter for urine collection and urine output monitoring during in-hospital patient management. The nursing staff was able to complete a total of 55 evaluations for a total of 44 catheter days. Evaluations were completed by a nurse or certified nurse assistant who placed and maintained the catheter. One evaluation was completed per staff member for the shift (day shift 7 AM to 7 PM and night shift 7 PM to 7 AM). The majority of the completed evaluations were during the day shift with some during the night shift. The mean age was 89 years. It was easy to apply (100%) and stayed in the majority (89%) of patients even during patient movements with one instance of urine leak (1.8%). None of the patients had any skin injury during the use or removal (0%). The staff felt the Acanthus condom catheter was better than current products in 95% of responses. Detailed results were shown in Table 1 and Table 2 with a summary of all responses in Table 3.

**Table 1:** Acanthus condom catheter nursing survey responses (patients 1-7)

Patient (age (in years))	Evaluation	Evaluation (day: 7 AM to 7 PM; night: 7 PM to 7 AM)	Easy to apply	Urine leak	Skin break	Better than others	Stays during patient movements	Pain or skin irritation or damage during removal	Comments	Catheter days
1 (57)	1	1/18/2024 day	Yes	No	No	Yes	Yes	No	Good adhesive; stayed in place	3
	2	1/18/2024 day	Yes	No	No	Yes	Yes	No	No urine leak	
	3	1/19/2024 day	Yes	No	No	Yes	Yes	No	No comments	
	4	1/21/2024 night	Yes	No	No	Yes	Yes	No	Durable and easy to apply	
2 (88)	5	1/28/2024 day	Yes	No	No	Yes	Yes	No	Adhesive prevents urine leak	2
	6	1/28/2024 day	Yes	No	No	Yes	Yes	No	No comments	
	7	1/29/2024 day	Yes	No	No	Yes	Yes	No	Adhesive prevents urine leak	
3 (67)	8	1/29/2024 day	Yes	No	No	No	No	No	No comments	3
	9	1/30/2024 day	Yes	No	No	Yes	Yes	No	No comments	
	10	1/31/2024 night	Yes	No	No	Yes	No	No	No comments	
4 (65)	11	2/4/2024 day	Yes	No	No	Yes	Yes	No	Easy to apply the adhesive; no leak	3
	12	2/4/2024 day	Yes	No	No	Yes	Yes	No	No leak during movements	
	13	2/4/2024 day	Yes	No	No	Yes	Yes	No	No urine leak during patient movements	
	14	2/5/2024 day	Yes	No	No	Yes	Yes	No	Although the patient was restless and moving, the catheter stayed on the patient's penis during the use	
	15	2/7/2024 night	Yes	No	No	Yes	No	No	Patient pulled out	
	16	2/7/2024 day	Yes	No	No	Yes	Yes	No	No comments	
5 (78)	17	2/24/2024 day	Yes	No	No	Yes	Yes	No	No comments	5
	18	2/26/2024 day	Yes	No	No	Yes	Yes	No	Patient pulled out	
	19	2/27/2024 night	Yes	No	No	Yes	No	No	No comments	
	20	2/27/2024 day	Yes	No	No	Yes	Yes	No	No comments	
	21	2/28/2024 day	Yes	No	No	Yes	Yes	No	No comments	
	22	2/28/2024 day	Yes	No	No	Yes	Yes	No	No comments	
	23	2/29/2024 day	Yes	No	No	Yes	Yes	No	Catheter stayed during patient movements	
6 (59)	24	3/29/2024 day	Yes	No	No	Yes	Yes	No	No leaking of urine	1
	25	3/29/2024 day	Yes	No	No	Yes	Yes	No	Drained urine; smaller penis	
7 (87)	26	4/4/2024 day	Yes	No	No	Yes	Yes	No	Easy to apply	3
	27	4/5/2024 day	Yes	No	No	Yes	Yes	No	NA	
	28	4/5/2024 night	Yes	Yes	No	Yes	No	No	Came off; penis is too small	
	29	4/6/2024 day	Yes	No	No	Yes	Yes	No	Stayed even if the patient is restless	

**Table 2:** Acanthus condom catheter nursing survey responses (patients 8-14)

Patient (age (in years))	Evaluation	Evaluation (day: 7 AM to 7 PM; night: 7 PM to 7 AM)	Easy to apply	Urine leak	Skin break	Better than others	Stays during patient movements	Pain or skin irritation or damage during removal	Comments	Catheter days
8 (76)	30	4/13/2024 day	Yes	No	No	Yes	Yes	No	Stayed good	3
	31	4/14/2024 day	Yes	No	No	Yes	Yes	No	No comments	
	32	4/15/2024 day	Yes	No	No	No	Yes	No	No comments	
9 (89)	33	5/4/2024 day	Yes	No	No	Yes	Yes	No	Stayed longer	2
	34	5/4/2024 day	Yes	No	No	Yes	Yes	No	Worked well	
	35	5/6/2024 day	Yes	No	No	Yes	Yes	No	Stayed	
10 (65)	36	5/16/2024 day	Yes	No	No	Yes	Yes	No	Very good stayed	5
	37	5/17/2024 day	Yes	No	No	Yes	Yes	No	NA	
	38	5/19/2024 day	Yes	No	No	Yes	Yes	No	Patient pulled out due to restlessness	
	39	5/20/2024 day	Yes	No	No	Yes	Yes	No	No comments	
	40	5/21/2024 day	Yes	No	No	Yes	Yes	No	No comments	
11 (61)	41	6/26/2024 day	Yes	No	No	Yes	Yes	No	Smaller penis; stays during patient movements	2
	42	6/29/2024 day	Yes	No	No	Yes	Yes	No	Smaller penis; stays well	
12 (81)	43	7/6/2024 day	Yes	No	No	Yes	Yes	No	Smaller penis	2
	44	7/7/72024 night	Yes	No	No	Yes	No	No	Patient pulled out	
13 (81)	45	7/12/2024 day	Yes	No	No	Yes	Yes	No	Smaller penis; stays in place	8
	46	7/13/2024 day	Yes	No	No	Yes	Yes	No	Adhesive keeps the catheter in place	
	47	7/13/2024 night	Yes	No	No	Yes	Yes	No	No comments	
	48	7/14/2024 day	Yes	No	No	Yes	Yes	No	No comments	
	49	7/15/2024 day	Yes	No	No	Yes	Yes	No	No comments	
	50	7/16/2024 day	Yes	No	No	Yes	Yes	No	Easy to apply	
	51	7/17/2024 day	Yes	No	No	Yes	Yes	No	Easy to put it on	
	52	7/18/2024 night	Yes	No	No	Yes	Yes	No	Very durable; long-lasting	
	53	7/19/2024 day	Yes	No	No	Yes	Yes	No	No skin issues	
14 (89)	54	7/10/2024 day	Yes	No	No	Yes	Yes	No	Needed for intake and output monitor	2
	55	7/11/2024 night	Yes	No	No	No	Yes	No	Durable	

**Table 3:** Summary of Acanthus condom catheter nursing survey responses

Total patients	Total evaluations (day+night)	Mean age (years)	Easy to apply	Urine leak	Skin break	Better than others	Stays during patient movements	Pain or skin irritation or damage during removal	Total catheter days
14	46+9=55	89	100%	1.80%	0%	95%	89%	0%	44

Out of six catheter slippages, four occurred during the night shift (7 PM to 7 AM) and one occurred during the day shift (7 AM to 7 PM). All of them were managed by placing the catheter back or replaced with a new one. During the course, the staff had learned to minimize catheter slippage by applying gentle pressure around the catheter under the rim or placing a few drops of silicone adhesive on the inner catheter wall under the catheter rim and applying gentle pressure. 

The data related to the length of time the catheter was in place for each patient was reported in Table 1 and Table 2 in the last column heading of catheter days, and all the data was summarized in Table 3 last column as total catheter days. For example, patient 5 and patient 10 had used the Acanthus condom catheter for five days, while patient 13 had it for eight days for external urinary collection. As a group, there were a total of 44 catheter days with an average of three days of use with very few patients having it for less than three days. The data was collected based on a nursing survey for each shift (12 hours ) either day or night shift. This is typically more than enough time for the catheter on the patient to see any impact on the skin. Data clearly showed that none of the 14 patients studied developed any skin redness or injury during the seven-month period.

## Discussion

Common problems in using condom catheters are finding the right size, frequent slippage, and time spent by nursing staff to frequently change to new ones. Placing an incorrect size can cause constriction of penile skin if smaller or cause urine leak if larger. Frequent catheter slippage and replacement with new catheters can potentially increase the cost, wastage, and risk of skin injury. Although nurses prefer condom catheters over indwelling urinary catheters for comfort and to reduce urinary tract infections, major drawbacks of catheter slippage and urine leak were still noted in one review of catheter preference by patients, and the staff felt that there was a need for developing more secure catheter that stays longer with no leak [3]. We believe that preventing frequent condom catheter slippages will help reduce the need for frequent catheter replacements; thus, the nurses can allocate their patient care time more efficiently. 

CAUTI due to indwelling urinary catheters contribute to 9% of all healthcare-associated infections [4]. Male external catheters play an important role in external urinary drainage as an alternative to invasive indwelling urinary catheters in nonobstructive patients for urine flow. They are used as an alternative to invasive urinary catheters to reduce the risk of CAUTI [5-7]. They are also used to maintain urinary continence and keep patients dry. The literature highlighted important problems related to condom catheter use, suggested opportunities to improve, and recommended the need for improvements to reduce frequent catheter slippage and leak. The other significant drawback of frequent usage of condom catheters is skin irritation and damage [8].

A reusable Acanthus condom catheter with a strap that goes around the patient's waist without using adhesive was previously clinically proof-tested and was published [9]. Skin irritation and damage have been reported using condom catheter use [10]. Many strategies were introduced to reduce CAUTI in hospital settings [11]. The Acanthus condom catheter with pressure-sensitive silicone adhesive was tested at both the bench- and bedside for safety and efficacy. It stayed on patients longer without urine leak in the majority even during patient movements. Medium-large sizes fit most patients even with varying penile sizes, which solved the important problem of picking the right size. Most importantly, the catheter was able to be pulled up and adjusted if noticed to be slipping and kept in place by applying gentle pressure with fingers around the catheter base under the rim or placing a few additional adhesive drops on the inner catheter wall under the catheter rim and applying gentle pressure. 

In a review of condom catheter use in spinal cord injury patients, it was emphasized the importance of proper fit, size selection, and proper seal. It is important to maintain skin hygiene, clean and remove urine residue buildup, and keep the foreskin over the glans penis in the uncircumcised penis to avoid skin breakdown [12]. In terms of frequency of changing the catheter, in a five-year study on spinal cord injury patients using the condom catheter, the authors did not find any statistically significant difference in skin irritation or damage when the catheter was changed daily versus every other day [13].

Another major challenge with condom catheters in general is selecting the correct size from different size options. The Acanthus condom catheter's ability to stretch can accommodate a wide range of penile sizes. The medium-large size (26-36 mm) was used in most of the patients (92%) with only one patient needing a small-medium size (18-25 mm) (8%). Because medium-large size was used in most patients, small-medium size was eliminated in the latter part of the study, and only the medium-large size was used even in smaller-sized penis with no urine leak probably due to the adhesive forming an effective seal around the catheter rim. Nurses also reported it was easy to place and keep it in place despite patient movements. Due to this clinical feedback, the medium-large size is the only size currently offered to fit 24-45-mm ranges. 

The current clinical proof testing demonstrated that the catheter was easy to apply (100%) and stayed in place during patient movements in the majority (89%). The Acanthus condom catheter stayed in most of the patients with some risk of coming out in restless and agitated patients with higher incidence during the night shift. The catheter was able to measure urine output to guide the patient's fluid management. Urine leak was reported in one patient during the night shift out of 55 responses (1.8%). A literature review showed about 15% of long-term condom catheter users develop skin irritation, inflammation, and skin injury [14]. However, the present review of 14 patients using the Acanthus condom catheter with a total of 44 catheter days over a span of seven months did not show any skin irritation, pain, or injury during the use or removal (0%). 

One other major drawback with condom catheters is frequent slippage due to penile retraction and loss of adhesive strength over a period of time, resulting in the slippage of the catheter and urine leak. The staff has a tendency to wrap using various methods to keep the catheter in place, most commonly using adhesive tape around the catheter and penis to prevent it from slipping which can increase the risk of penile strangulation and injury. We believe that the Acanthus condom catheter's ability to expand with changing penile size and soft, smooth, and rounded inner catheter wall with pressure-sensitive silicone adhesive keeps the catheter in place without requiring additional supportive tape or straps. 

**Figure 8:**
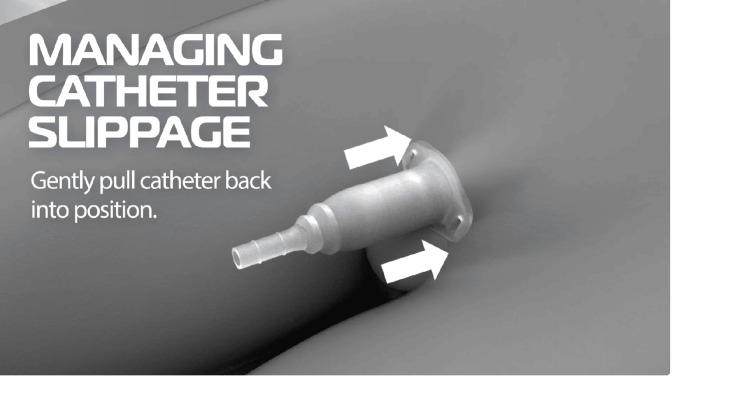
Pulling back Acanthus condom catheter during slippage Image Credit: Author

**Figure 9:**
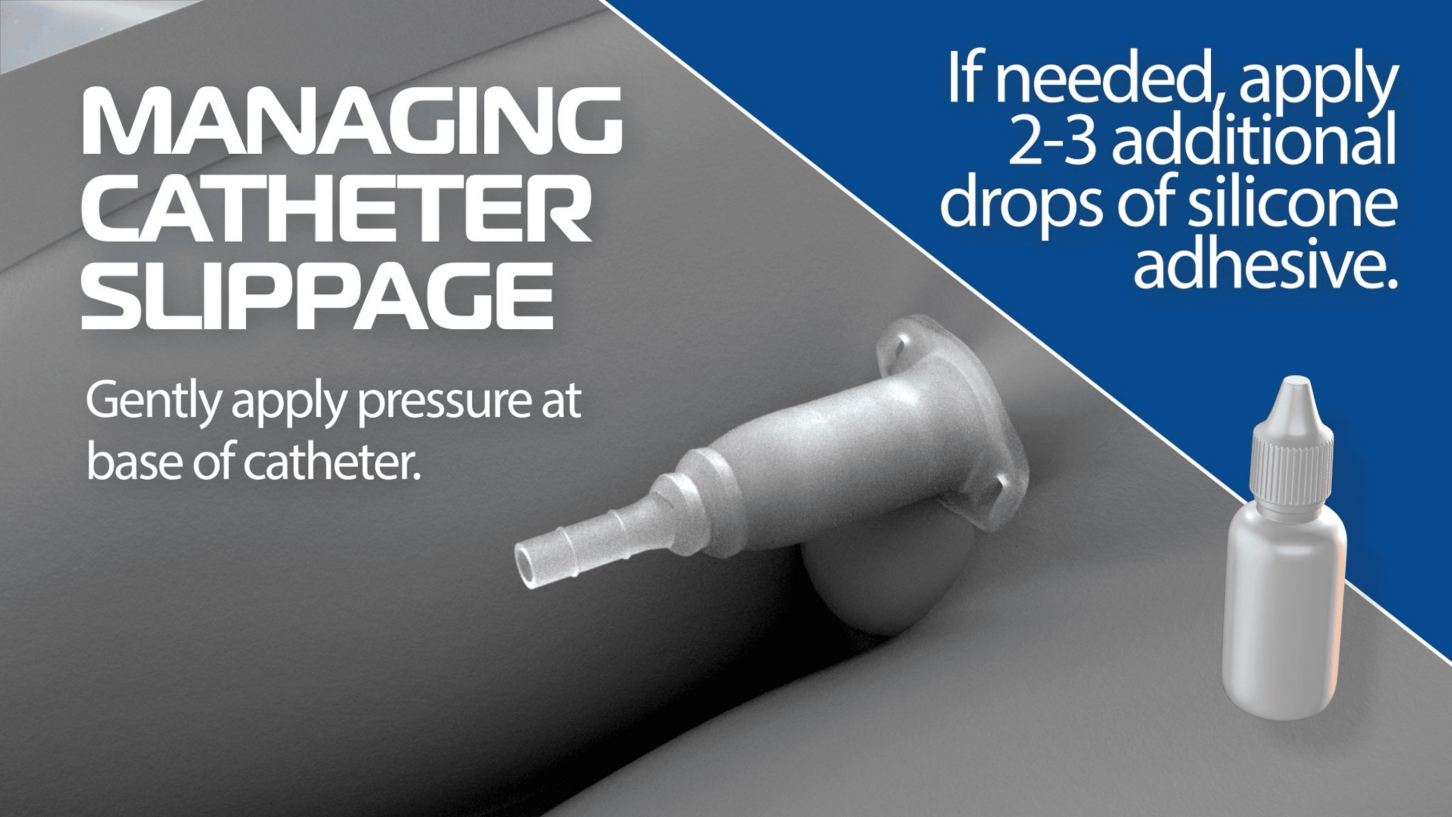
Managing Acanthus condom catheter slippage by adding additional adhesive Image Credit: Author

Nurses report that condom catheters frequently slip out due to moisture and penile retraction with the catheter barely holding on to the penile skin tip and often balloon out and slip with rapid urine flow during bladder emptying. They need to be replaced with a new one every time the catheter comes out. The Acanthus condom catheter can be adjusted upwards by pulling up on the penile shaft and can be kept in place by applying gentle pressure under the catheter rim (Figure 8). If needed, adding a few drops of silicone adhesive to the inner catheter wall under the catheter rim and applying gentle finger pressure can restore the catheter in position (Figure 9).

If the Acanthus condom catheter completely comes out accidentally by the patient pulling out or during patient movements, the same catheter can be placed back after washing if needed on a daily basis and can be reused for up to three days.

Another precaution to consider when using condom catheters in general is to make sure the urine flows freely from the catheter to the drainage bag at the bedside avoiding any twists or kinks at the catheter tip. Lift the drainage tube from the patient's side to improve urine flow into the drainage bag, and empty the drainage bag often to reduce the backflow. Another measure to clear the urine from stagnating in the drainage tube is to disconnect it from the catheter tip and lift it towards the drainage bag periodically to clear the urine and keep the penile skin dry to prevent excess moisture-related skin injury. With the Acanthus condom catheter being thicker and pliable, the catheter tip can be maneuvered to reduce any twists and kinks to allow free urine flow. In the current clinical series, none of the patients had any issues with the urine flow and drainage into the bag. Nurses were educated and instructed on how to keep the penile skin clean and dry as much as possible.

CAUTI is one of the most common healthcare-associated infections in the United States. The Centers for Medicare and Medicaid Services stopped reimbursing hospitals for the costs of caring for patients who develop CAUTI and its related complications such as bacteremia and sepsis [15]. Due to this pressure, hospitals adopted strict policies and guidelines on the discontinuation of indwelling urinary catheters to replace them with external catheters or other means of voiding such as urinals once the patient is able to maintain continence.

Although the current study did not look into the cost, based on the clinical feedback, condom catheters needed to be replaced with new ones at least one or two times or more per day requiring many catheters needed for three days of use adding up the costs. The Acanthus condom catheter being adjustable when slipping and reusable for three days could potentially reduce the number of catheters used per patient per day which could reduce the cost and wastage. 

The present study was conducted as part of a quality improvement project looking into the safety and efficacy of the Acanthus condom catheter. The limitations of the current study are the small sample size and no comparison with other products in the market. Moreover, the study could also have a subjective bias due to answering prepopulated questions on the survey although some users left comments on the product's effectiveness and shared their experience when compared to using other products in the market. Future large-scale studies would be helpful to answer the cost-effectiveness of the catheter. 

## Conclusions

The present case series demonstrated that the Acanthus condom catheter is safe and effective in draining urine and can be used as an alternative to indwelling urinary catheters in patients with no urinary obstruction. Medium-large size fits most due to elasticity and stretch dimension reducing the burden of selecting the correct size from multiple size options. Catheter slippage was managed well by applying gentle pressure around the catheter under the rim or applying a few drops of silicone adhesive to the inner catheter wall under the rim and applying gentle pressure. The catheter can be reused for up to three days if washed daily. The Acanthus condom catheter did not cause any skin irritation or damage. The reusable and adjustable feature of the Acanthus condom catheter could potentially reduce the cost and wastage of the catheters and could potentially improve nursing work-time efficiency. Future prospective studies would be needed to confirm these findings and to see if the Acanthus condom catheter is cost-effective for external urinary drainage in males. 

## Figures and Tables

**Figure 10:**
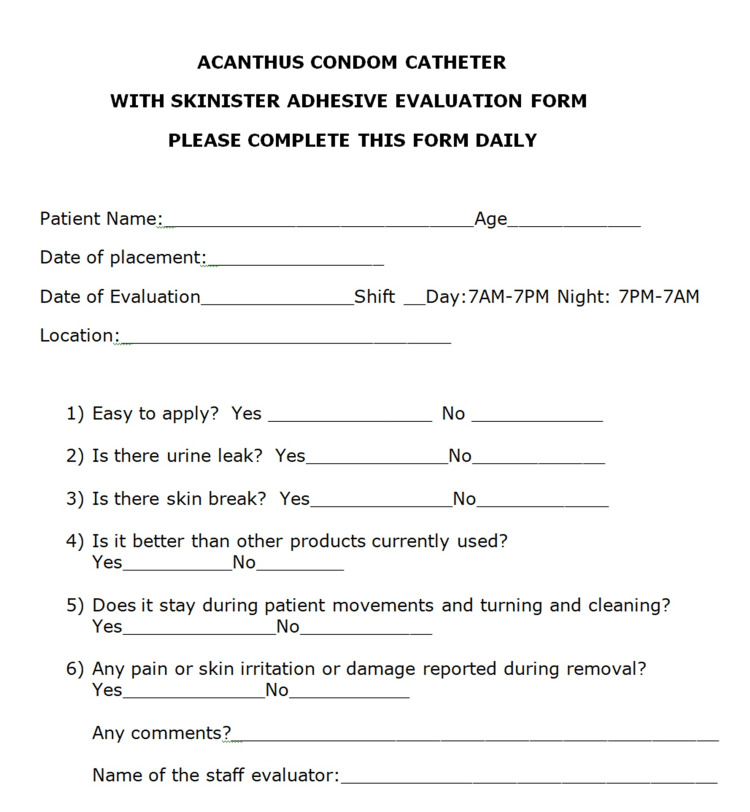
Nursing survey questionnaire
